# The Resolution of Periodontal Inflammation Promotes Changes in Cytokine Expression in the Intestine and Gingival Tissues of Aged Rats with DSS-Induced Colitis

**DOI:** 10.3390/jcm12134326

**Published:** 2023-06-27

**Authors:** João Martins de Mello-Neto, Edilson Ervolino, Gayathiri Elangovan, Luan Felipe Toro, Jaehee Lee, Anders Gustafsson, Carlos Marcelo da Silva Figueredo

**Affiliations:** 1 College of Medicine and Dentistry, James Cook University, Cairns, QLD 4870, Australia; joao.martinsdemelloneto@jcu.edu.au; 2 School of Medicine and Dentistry, Griffith University, Brisbane, QLD 4111, Australia; gayathiri.elangovan@griffithuni.edu.au (G.E.); rachel.lee2@griffithuni.edu.au (J.L.); 3 Department of Basic Sciences, School of Dentistry, São Paulo State University-UNESP, Araçatuba 16015-050, SP, Brazil; e.ervolino@unesp.br (E.E.); luan.toro@unesp.br (L.F.T.); 4 Division of Oral Diseases, Department of Dental Medicine, Karolinska Institutet, 171 77 Stockholm, Sweden; anders.gustafsson@ki.se

**Keywords:** cytokines, intestine, rats, periodontitis, dextran sulphate sodium, inflammatory bowel diseases

## Abstract

Our research aimed to explore how resolving periodontal inflammation impacts cytokine expression in the colons of aged Wistar rats. Research studies involving animals have been conducted to investigate the two-way relationship between periodontitis and inflammatory bowel disease (IBD), where chronic inflammation in either the mouth or intestines can negatively affect the other. We allocated seventeen male Wistar rats aged between 8 and 11 months to one of four groups: (1) ligature-induced periodontitis (LIP) without the resolution of periodontal inflammation (RPI) (LIP; n = 4), (2) LIP + RPI (n = 4), (3) LIP + dextran-sulphate-sodium-induced colitis (DIC) without RPI (n = 4), and LIP + DIC + RPI (n = 5). We performed histopathological and immunological analyses on periodontal and intestinal tissues and analysed cytokine expressions using a Rat Cytokine 23-Plex Immunoassay. Our findings showed that animals with and without DIC who underwent RPI showed significantly lower levels of IL-2, IL-4, IL-5, IL-10, IL-13, IL-17, IL-18, and TNF-α in the intestine compared to those without treatment. The RPI effectively reduced the number of inflammatory cells in the lamina propria and restored the epithelial barrier in the intestine in animals with DIC. The resolution of periodontal inflammation significantly reduced the levels of pro-inflammatory cytokines and chemokines in the intestines of aged rats with and without DSS-induced colitis.

## 1. Introduction

Periodontitis has been associated with several systemic diseases, most notably diabetes mellitus, cardiovascular diseases, adverse pregnancy outcomes, and inflammatory bowel disease (IBD) [1]. Moreover, it has been shown that individuals with IBD are more likely to have periodontitis than those without IBD [2].

Animal models have been used to elucidate the possible mechanisms linking periodontitis and IBD [3,4,5]. Kitamoto et al. (2020) reported that oral pathobiont-reactive T cells could migrate from the oral mucosa through the lymphatics to the gut during ligature-induced periodontitis. Once in the guts, such specific cell groups can be activated by the ectopically colonising oral pathobionts, exacerbating colitis mainly by transmigrated Th17 cells of oral origin [3]. More recently, Yuan et al. (2022) showed that ligature-induced periodontitis significantly elevates Th1 and Th17 cells in submandibular lymph nodes and the proportion of circulating Th1, Th2, and Th17 cells in peripheral blood, suggesting that periodontitis increases the proportion of pro-inflammatory Th-cell subsets locally and systemically [6].

Our group has previously shown that dextran sulphate sodium (DSS)-induced colitis (DIC) significantly increases the expression of IL-1α, IL-1β, IL-2, IL-6, IL-12, IL-13, GM-CSF, IFN-γ, and TNF-α in the gingival tissues of Wistar rats [5]. Further experiments showed that the ligature-induced periodontitis led to an overexpression of Th1/Th2-related cytokines in the intestines of Wistar rats [7]. Although evidence of an oral–intestine interplay is apparent when using an animal model, the effect of the resolution of periodontal inflammation is yet to be clarified. If the resolution of the periodontal inflammation leads to a decrease in intestinal inflammation, this would indicate that thorough periodontal treatment could be of value for patients with IBD.

Huang et al. (2020) used a mice model to demonstrate that ligature-induced periodontitis significantly compromises the intestinal barrier and that the composition of the intestinal microbiome can be significantly altered after non-surgical periodontal treatment (NSPT). The authors also pointed out that animals submitted to NSPT tended to restore the intestinal barrier, although these differences were not significant [8]. However, the effect of the resolution of periodontal inflammation on intestinal tissues’ expression of pro- and anti-inflammatory cytokines needs further investigation. We used a 23- cytokine multiplex panel to investigate the effect of the resolution of periodontal inflammation on the expression of cytokines in the colons of aged Wistar rats with DSS-induced colitis. 

## 2. Materials and Methods

### 2.1. Animals

In this study, seventeen male rats aged between 8 and 11 months and weighing between 460 and 675 g were used. The rats were of the Wistar–Rattus norvegicus breed and were supplied by the Animal Resources Centre (Harrison Road, Forrestfield, Western Australia). Each rat was housed individually in a cage for 2 weeks before the experiment began. During this period, they were acclimatised to a 12 h light/dark cycle and were provided with food and water ad libitum, which was continued throughout the experimental period. The rats were divided randomly into four groups: (1) LIP (ligature-induced periodontitis) (n = 4), (2) LIP + RPI (resolution of periodontal inflammation) (n = 4), (3) LIP + DIC (DSS-induced colitis) (n = 4) and (4) LIP + DIC + RPI (n = 5).

During procedures that could involve pain or discomfort, such as ligature installation and euthanasia, the animals were given 4% atmospheric isoflurane through inhalation, with an oxygen flow rate of 400 mL/min at the induction chamber. To ensure their comfort, the isoflurane level was subsequently reduced to 1–2%, with an oxygen flow rate of 200 mL/min to the animal’s nose. Throughout the procedure, the animal’s condition and signs of pain were closely monitored, and records were kept on formal score sheets. This research followed the guidelines of ARRIVE (Animal Research: Reporting of In Vivo Experiments) [9].

### 2.2. Ligature-Induced Periodontitis

**Figure 1:**
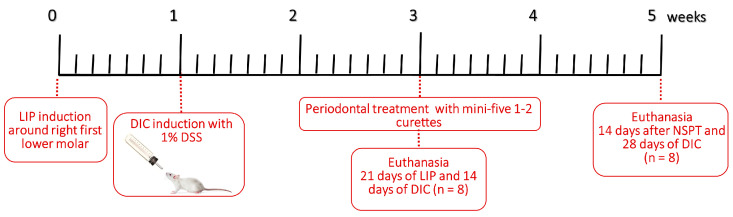
Study design. DIC: DSS-induced colitis; DSS: dextran sulphate sodium; LIP: ligature-induced periodontitis; NSPT: non-surgical periodontal treatment.

The right first mandibular molar was surrounded by a sterile braided multifilament surgical silk ligature 5-0 (SMI Suture, Steinerberg, St. Vith, Belgium) to induce periodontitis. Following the placement of ligatures, the animals were placed on a heated pad to aid their recovery. Rats that were unconscious or that had undergone the procedure were kept in separate cages from the other active rats to prevent any unnecessary stress. The ligatures were left in place for 21 days to induce LIP, as shown in Figure 1 [10]. Throughout the experimental period, the ligature’s presence was monitored every other day. Plaque build-up was observed in the ligatures, while the gingival tissues appeared red and swollen. One animal from LIP + DIC + RPI lost its ligature and was excluded from the present study. 

### 2.3. Induction of DSS-Induced Colitis (DIC)

This study utilized the DIC model, which involved the application of 1% DSS (MW: 36,000–50,000 Da; MP Biochemicals, Shanghai, China) in the drinking water for 14 days in the LIP + DIC group and 28 days in the LIP + DIC + RPI group provided ad libitum to the animals as shown in Figure 1 [11]. To ensure the accurate monitoring of disease activity, animals from the LIP + DIC and LIP + DIC + RPI groups were closely observed three times a week for stool consistency, weight loss, and bleeding.

### 2.4. Resolution of Periodontal Inflammation

To achieve a comprehensive resolution of periodontal inflammation, the ligated molars’ root surfaces were manually cleaned using mini-five 1–2 curettes (Hu-Friedy^®^, Rockwell St., Chicago, IL, USA) after removing the ligatures. The periodontal debridement was performed by a skilled operator (JMMN) who meticulously executed 10 distal-mesial traction movements on both the buccal and lingual surfaces of the molar. This ensured the removal of all the accumulated debris and plaque from the root surfaces, promoting optimal healing and preventing further oral inflammation [12]. 

### 2.5. Clinical Assessment

During the experiment, rats’ body weight, stool characteristics, and the presence of blood were monitored every 3 days. We utilized the ‘NDC Pro Advantage Faecal Occult Blood’ clinical kits (NDC, Inc. 402 BNA Drive, Suite 500, Nashville, TN, USA) to detect occult blood. The results were scored as 0 (no colour development), 1 (fleck of colour reaction), 2 (consistent blue colour), 3 (rust-coloured stools + blue reaction), or 4 (the presence of wet blood + dark blue reaction). To calculate the disease activity index (DAI), we added the scores for weight loss, stool traits, and blood in the stool, and divided the result by 3. DAI scores were determined based on weight changes, occult blood presence, and stool form. Weight changes were scored as 0 for no change, 1 for a change of less than 5%, 2 for a change of 5% to 10%, 3 for a change of 11% to 15% and 4 for a change of 16% or higher. Occult blood was scored as 0 for negative, 2 for positive, and 4 for gross bleeding. Stool form was scored as 0 for normal, 2 for loose stools, and 4 for diarrhea.

### 2.6. Sample Collection and Euthanasia

After 21 days of ligature placement, euthanasia was conducted in both the LIP and LIP + DIC groups. In order to assess the effects of inflammation resolution, animals from the LIP + RPI and LIP + DIC + RPI groups underwent euthanasia 14 days after ligature removal and non-surgical periodontal treatment. The animals were deeply anesthetised and euthanised through cervical dislocation. Hemimandibles and biopsy samples from the gingiva and intestine were gathered for histological and immunological analysis.

### 2.7. Cytokine Analysis

Using a scalpel blade 15c, the gingival tissue from the lingual site surrounding the mandibular first molar was surgically removed. Next, we accurately measured the weight of the removed tissues using an analytical balance (Ohaus, Parsippany, NJ, USA) and then transferred them to a microtube containing two ultrapure 3.0 mm zirconia beads, 300 μL of phosphate-buffered saline (PBS, Sigma-Aldrich, St-Louis, MO, USA), and 50 μL of protease inhibitor (Sigma-Aldrich, St. Louis, MO, USA). To homogenize the tissue, a cell disruptor (TissueLyser II, QIAGEN, Chadstone, VIC, Australia) was used at 30 Hz for 4 min. After being homogenized, the mixture was centrifuged at 10,000 rpm for 10 min. Following this, the resulting supernatant was stored at a temperature of −80 °C until it could be analysed using the Bio-Plex Pro Rat Cytokine 23-Plex Immunoassay (Analytes: G-CSF, GM-CSF, GRO/KC, IFN-γ, IL-1α, IL-1β, IL-2, IL-4, IL-5, IL-6. IL-7, IL-10 IL-12 (p70), IL-13, IL-17A, IL-18, M-CSF, MCP-1, MIP-1α, MIP-3α, RANTES, TNF-α, and VEGF) to evaluate the level of cytokines and chemokines known to be related to the pathogenesis of both IBD and periodontitis. 

### 2.8. Processing for Histology

The hemimandibles underwent demineralization in 10% ethylenediamine tetraacetic acid (EDTA) (Chemical^®^ Sigma) in PBS for 60 days. They were then subjected to traditional histological procedures for paraffin embedding. The hemimandibles were gathered and preserved in 4% formaldehyde for 48 h. Sections measuring 4 µm thick were sliced from the vestibular to the lingual and stained with hematoxylin-eosin (H&E) for histopathological and histometric analyses. Intestinal biopsies were also obtained and preserved in 4% formaldehyde for 72 h before undergoing conventional histological processing for paraffin embedding. 

### 2.9. Histopathological Evaluation

The H&E slides were scanned using an Olympus VS200 Research Slide Scanner. QuPath 0.3.0 and Image J software were employed to analyse the results. The histopathological assessments were undertaken by a certified histologist (EE). To ensure impartiality, the histologist was blinded to the groups and calibrated before beginning the assessments. The evaluation focused on various aspects such as the intensity and extent of the inflammatory response, alveolar bone resorption, cellular pattern, and connective tissue structure, as well as the structuring of the alveolar and intestinal mucosa.

### 2.10. Histometric Analysis

To determine alveolar bone loss, the distance between the bone crest and cementum-enamel junction (CEJ) on the mesial of the lower right first molar was measured. This was done using QuPath 0.3.0 and Image J software. To ensure accuracy, the same examiner performed the measurements three times on different days in the same specimen. 

### 2.11. Statistical Analysis

To analyse the data, SPSS 21.0 (IBM, Armonk, NY, USA) was used. Data normality was assessed with the Kolmogorov–Smirnov test. Medians with interquartile ranges were used to present continuous variables. For histometric comparisons, we performed an analysis of variance (ANOVA), followed by the Tukey post-test. Semi-quantitative data from histological analyses underwent Shapiro–Wilk variance analysis, and a Kruskal–Wallis test was followed by the Student–Newman–Keuls post-test. Immunological analyses were analysed using Mann–Whitney tests to compare continuous variables between groups. We considered statistical significance for *p* ≤ 0.05. 

## 3. Results

### 3.1. Clinical Assessment

**Figure 2:**
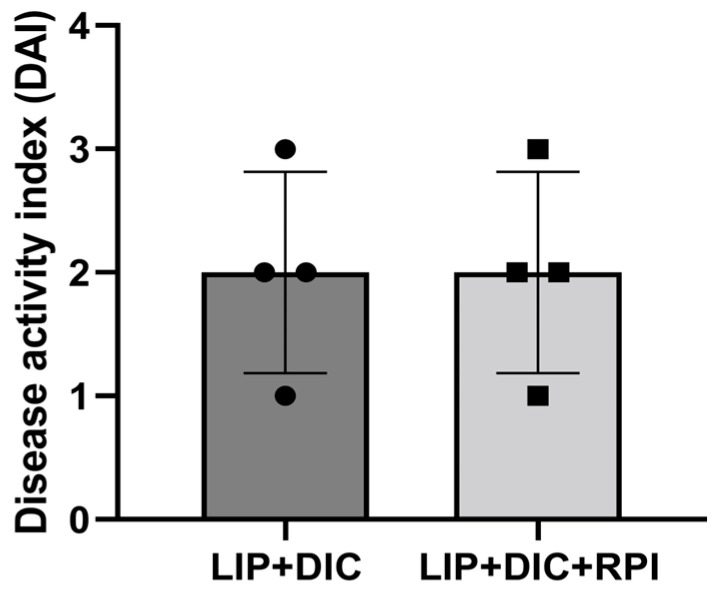
Disease activity index (DAI). DIC: DSS-induced colitis; LIP: ligature-induced periodontitis; RPI: resolution of periodontal inflammation.

**Figure 3:**
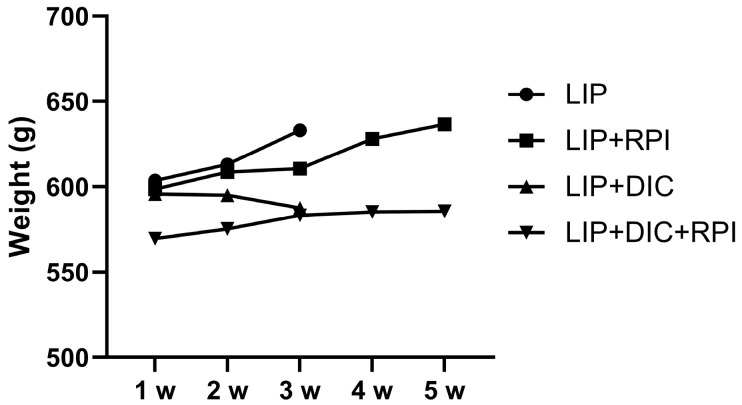
Graph showing animals’ body weight throughout the experimental period. LIP: ligature-induced periodontitis; LIP + DIC: ligature-induced periodontitis with DSS-induced colitis; RPI: resolution of periodontal inflammation; µm: micrometres.

Two weeks after administering DSS, clinical signs of DIC, including weight loss and stool bleeding, started to manifest. There was no discernible variation in DAI between the LIP + DIC and LIP + DIC + RPI groups. One animal from the LIP + DIC group and one from the LIP + DIC + RPI group presented a DAI of 3, two from each group presented a DAI of 2, and one from each group presented a DAI of 1 (Figure 2). Animals from the LIP (initial weight median: 609.5 g; STD: 49.1 g) and LIP + RPI ( initial weight median: 608.5 g; STD: 67 g) groups presented a weight gain of 4.8% and 4.6%, respectively. The animals from the LIP + DIC (initial weight median: 591 g; STD: 32.9 g) and LIP + DIC + RPI (initial weight median: 543.5 g; STD: 75.27 g) groups presented a weight loss of 0.6% and weight gain of 1.6%, respectively. There was no significant difference in weight among the groups (Figure 3). 

### 3.2. Histopathological and Histometric Analyses

#### 3.2.1. Ligature-Induced Periodontitis

**Figure 4:**
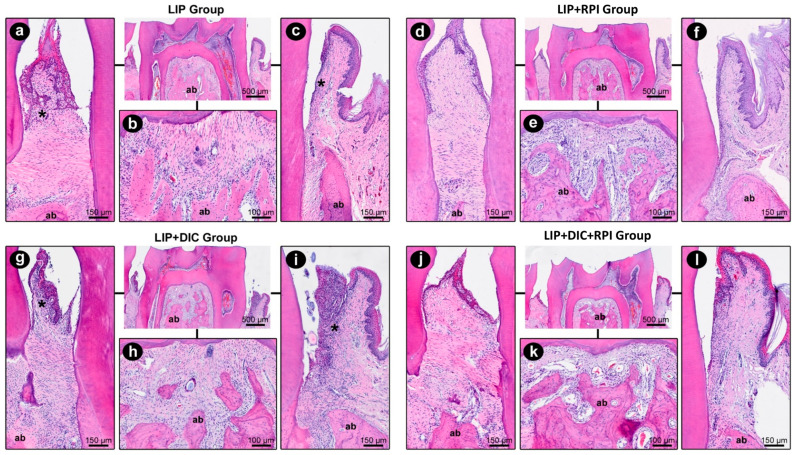
Photomicrographs of the right mandibular first molar showing the course of the inflammatory response in the distal (**a**,**d**,**g**,**j**), furcation (**b**,**e**,**h**,**k**), and mesial (**c**,**f**,**i**,**l**) areas at 21 days (LIP and LIP + DIC) and 35 days (LIP + RPI and LIP + DIC + RPI). ab: alveolar bone; DIC: DSS-induced colitis; *: inflammatory infiltrate; LIP: ligature-induced periodontitis; NSPT: non-surgical periodontal treatment. Scale bars: 500 µm, 150 µm, and 100 µm. Staining: hematoxylin and eosin (H&E).

Ligature removal and non-surgical periodontal treatment resulted in a decrease in local inflammation and a reduction in tissue degradation in the LIP + RPI and LIP + DIC + RPI groups. The LIP + DIC group showed a more intense local inflammatory response and a more significant impairment of the periodontal tissue structure than the LIP group. In Table 1, the parameters, scores, and distribution of specimens are displayed based on their histopathological analysis of periodontal tissues in the LIP, LIP + DIC, LIP + RPI, and LIP + DIC + RPI groups. Figure 4 depicts the histopathological characteristics of the experimental groups in the first mandibular right molar’s distal, furcation, and mesial areas.

**Figure 5:**
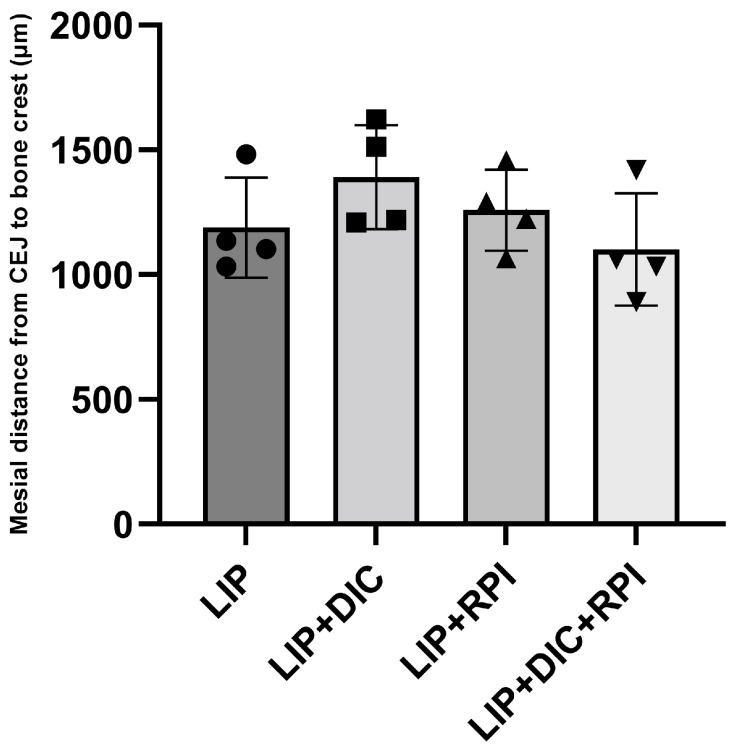
Graphic displaying the average and standard deviations of the linear distance from CEJ to the alveolar bone crest measured in micrometres (µm) in the mesial, furcation, and distal regions. CEJ: cementum enamel junction; LIP: ligature-induced periodontitis; LIP + DIC: ligature-induced periodontitis with DSS-induced colitis; RPI: resolution of periodontal inflammation; µm: micrometres.

The histometric analyses showed no significant differences between the groups (Figure 5).

**Figure 6:**
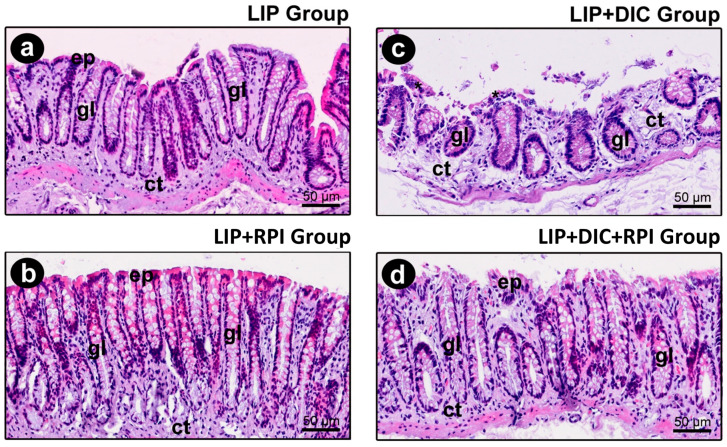
Photomicrographs of the intestine of the LIP (**a**), LIP + RPI (**b**), LIP + DIC, (**c**), and LIP + DIC + RPI (**d**) groups. ep: intestinal epithelium; ct: connective tissue; gl: glands; *: inflammatory infiltrate. Scale bars: 50 μm. Staining: hematoxylin and eosin (H&E).

The epithelium of the intestinal mucosa was intact in the LIP and LIP + RPI groups. The lamina propria comprised loose connective tissue with fibroblasts and a few inflammatory cells, predominantly mononucleated (Figure 6a,b).

#### 3.2.2. DSS-Induced Colitis

In the LIP + DIC group, the epithelium of the intestinal mucosa was discontinuous. In such regions, an exposure of the lamina propria was observed, which consisted of loose connective tissue with a moderate number of inflammatory cells (predominantly consisting of mononucleated cells) (Figure 6c). In the LIP + DIC + RPI group, the epithelium showed some small foci of discontinuity, which were more sparsely distributed. The lamina propria comprised loose connective tissue with few inflammatory cells (Figure 6d). In the LIP, LIP + RPI, and LIP + DIC + RPI groups, the structure of the intestinal glands was little affected (Figure 6a,b,d). In the LIP + DIC group, the structure of the intestinal glands was preserved but more sparsely distributed (Figure 6c). Table 2 shows specimens’ parameters, scores, and distribution according to the histopathological analysis of intestinal tissues in the LIP, LIP + DIC, LIP + RPI, and LIP + DIC + RPI groups.

### 3.3. Immunological Analyses

#### 3.3.1. Gingival Cytokine Expression

**Figure 7:**
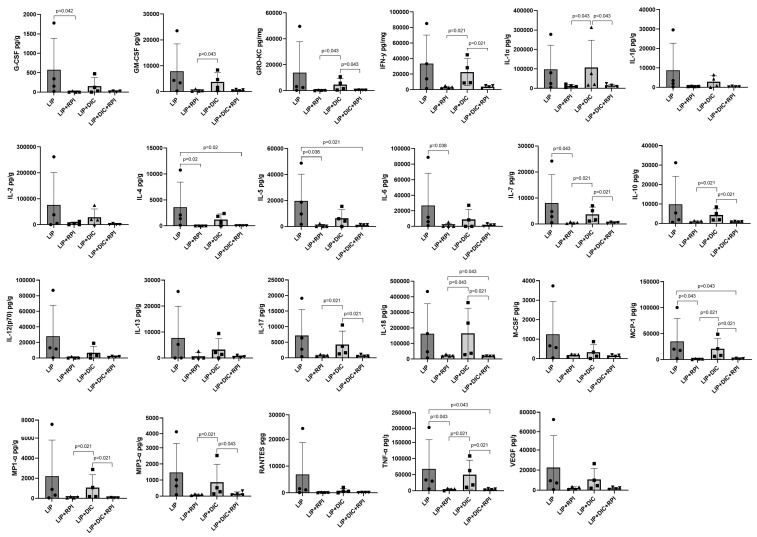
Levels of G-CSF, GM-CSF, GRO-KC, IFN-γ, IL-2IL-1α, IL-1β, IL-2, IL-4, IL-5, IL-6, IL-7, IL-10, IL-12 (p70), IL-13, IL-17, IL-18, MCP-1, M-CSF, MIP1-α, MIP3-α, RANTES, TNF-α, and VEGF in the gingival tissues of ligature-induced periodontitis (LIP) and LIP associated with the resolution of periodontal inflammation (LIP + RPI); LIP + DSS-induced colitis (DIC) without RPI (n = 4); LIP + DIC + RPI (Mann–Whitney test). G-CSF: Granulocyte colony-stimulating factor; GM-CSF: Granulocyte-macrophage colony-stimulating factor; GRO-KC: Keratinocyte chemoattractant (KC)/growth-regulated oncogene (GRO); IFN: interferon; IL- interleukin; MCP-1: Monocyte chemoattractant protein-1; M-CSF: Macrophage colony-stimulating factor; MIP-1α: Macrophage inflammatory protein-1 alpha; MIP3-α: Macrophage inflammatory protein-3 alpha; RANTES: Regulated on activation, normal T cell expressed and secreted; TNF: Tumor necrosis factor; VEGF: Vascular endothelial growth factor.

The LIP + DIC + RPI group presented a significantly lower expression of GM-CSF, GRO-KC, INF-γ, IL-1α, IL-7, IL-10, IL-17, IL-18, MCP-1, MP1-α, MIP3-α, and TNF-α compared to the LIP + DIC group. The LIP + RPI group presented a significantly lower expression of G-CSF, IL-4, IL-5, IL-6, IL-7, MCP-1, and TNF-α than the LIP group (Figure 7). No noteworthy variances were observed in cytokine expression between the LIP and LIP + DIC groups or between the LIP + RPI and LIP + DIC + RPI groups. 

#### 3.3.2. Intestinal Cytokine Expression

**Figure 8:**
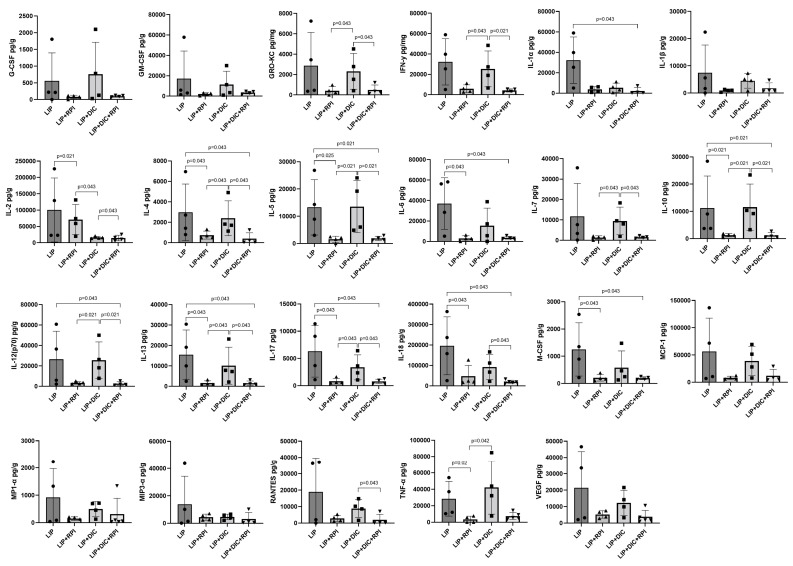
Levels of G-CSF, GM-CSF, GRO-KC, IFN-γ, IL-2IL-1α, IL-1β, IL-2, IL-4, IL-5, IL-6, IL-7, IL-10, IL-12 (p70), IL-13, IL-17, IL-18, MCP-1, M-CSF, MIP1-α, MIP3-α, RANTES, TNF-α, and VEGF in the intestines of ligature-induced periodontitis (LIP) and LIP associated with the resolution of periodontal inflammation (LIP + RPI); LIP + DSS-induced colitis (DIC) without RPI (n = 4); LIP + DIC + RPI (Mann–Whitney test). G-CSF: Granulocyte colony-stimulating factor; GM-CSF: Granulocyte-macrophage colony-stimulating factor; GRO-KC: Keratinocyte chemoattractant (KC)/growth-regulated oncogene (GRO); IFN: interferon; IL- interleukin; MCP-1: Monocyte chemoattractant protein-1; M-CSF: Macrophage colony-stimulating factor; MIP-1α: Macrophage inflammatory protein-1 alpha; MIP3-α: Macrophage inflammatory protein-3 alpha; RANTES: Regulated on activation, normal T cell expressed and secreted; TNF: Tumor necrosis factor; VEGF: Vascular endothelial growth factor.

The LIP + RPI group showed significantly lower expressions of IL-2, IL-4, IL-5, IL-6, IL-10, IL-13, IL-17, IL-18, M-CSF, and TNF-α compared to the LIP group. The LIP + DIC + RPI group showed significantly lower levels of GRO-KC, IFN-γ, IL-2, IL-4, IL-5, IL-7, IL-10, IL-12 (p70), IL-13, IL-17, IL-18, and RANTES compared to the LIP + DIC group (Figure 8). The LIP and LIP + DIC groups and the LIP + RPI and LIP + DIC + RPI groups did not show any significant differences when compared.

## 4. Discussion

Our study showed that the resolution of ligature-induced periodontal inflammation by ligature removal together with manual scaling decreased intestinal inflammation in animals with DSS-induced colitis. The inflammation reduction was shown by lower levels of pro-inflammatory cytokines associated with lower numbers of inflammatory cells in the lamina propria and signs of epithelial barrier restoration in the intestine. To the best of our knowledge, our study is the first to indicate that periodontal treatment may have a beneficial impact on cytokine expression in intestinal tissue. Huang et al. (2020) investigated the impact of non-surgical periodontal treatment on the disturbed gut microbiome in mice. According to the authors, non-surgical periodontal treatment showed a tendency to restore the gut microbiota to normal levels, and, like our findings, an improvement in the intestinal mucosal barrier impaired by periodontitis was also observed [8]. Taken together, both studies support the hypothesis that periodontal treatment might improve the intestinal conditions of patients with inflammatory bowel diseases.

We also showed that aged rats with DIC receiving periodontal treatment presented reduced intestinal IL-17 and IFN-γ compared to non-treated animals. Kitamoto et al. (2020) have demonstrated that ligature-induced periodontitis worsens intestinal inflammation in mice with DIC. This is due to an increase in immune infiltration into the gut lamina propria, including Th17 subsets, B cells, and γδ T cells [3]. In addition, their findings revealed that T cells in the colons of mice with LIP + DIC produced higher levels of IL-17A and IFN-γ compared to T cells from mice with only DSS-induced colitis or non-colitis control mice., suggesting that ligature-induced periodontitis exacerbates DIC due to an accumulation of Th17 and Th1 cells [3]. We have previously shown increased levels of Th1/Th2-related cytokines and inflammatory cells in rats with LIP in the intestine [7]. Thus, periodontal treatment may downregulate the gut immunological response induced by the presence of periodontal disease by reducing the expression of Th1- and Th17-related cytokines. 

Our histopathological results showed that periodontally treated aged rats presented lower numbers of inflammatory cells in the lamina propria and signs of epithelial barrier restoration in the intestine. As mentioned before, according to Huang et al. (2020), non-surgical periodontal treatment was found to improve the intestinal barrier in mice models [8]. Intestinal barrier defects have been associated with several diseases, such as IBD, colon carcinoma, and celiac disease [13]. Taken together, these findings reinforce the possible role of periodontal treatment in restoring intestinal epithelial layer integrity. Additionally, the histopathological analyses showed more damage to the bone structure in the LIP group. On the other hand, the histometric quantitative analyses of the teeth’s mesial aspect showed no significant differences in bone loss. The reason for this discrepancy is unknown. We speculate that although the bone level in the LIP + RPI group improved, it could not be quantified in a histometric analysis.

Besides the intestinal impact, the periodontal treatment significantly reduced cytokine expression in gingival tissues. Our previous publication aligns with these findings. We have shown a significant increase in the expression of Th1/Th2-related cytokines in the gingival tissues of rats with ligature-induced periodontitis [5]. Moreover, the periodontal tissue of aged rats that received periodontal treatment presented histopathological features compatible with health. 

Our study used aged animals (8–11 months old) aiming to mirror adults of approximately 30 years old, making our model suitable to experimentally evaluate LIP and DIC [14]. It has been shown that individuals between the ages of 30 and 45 are more prone to severe and widespread periodontitis, with its onset commonly occurring between the ages of 22 and 28 [15]. Regarding IBD, it has been established that both UC and CD have the highest incidence rate among individuals aged 15 to 29 years old [16]. Therefore, we believe that aged rats better reproduce the clinical conditions observed in human studies. On the other hand, differences in anatomy, physiology, developmental biology, and age must be taken into consideration when analysing our results. 

Our study highlights the importance of clinical periodontal intervention to better understand the bidirectional interactions between periodontitis and inflammatory bowel disease. However, this study has limitations, such as the small sample size and short evaluation period. In addition, our study did not evaluate gut and oral microbiota, which might have had a critical impact on the outcomes. Thus, our results should be interpreted with caution.

## 5. Conclusions

The resolution of periodontal inflammation significantly reduced levels of pro-inflammatory cytokines and chemokines in the intestines of aged rats with and without DSS-induced colitis.

## Figures and Tables

**Table 1:** The parameters, scores, and distribution of specimens based on the histopathological analysis of periodontal tissues in the lower first molar region of the experimental groups.

PARAMETERS AND RESPECTIVE SCORES	% of Animals			
	EXPERIMENTAL GROUPS			
	LIP	LIP + RPI	LIP + DIC	LIP + DIC + RPI
INTENSITY OF LOCAL INFLAMMATORY INFILTRATE				
(1) absence of inflammation	-	50%	-	50%
(2) small quantity of inflammatory cells (1/3 of cells were inflammatory cells)	-	50%	-	50%
(3) moderate quantity of inflammatory cells (1/3 to 2/3 were inflammatory cells)	100%	-	25%	-
(4) large quantity of inflammatory cells (more than 2/3 were inflammatory cells)	-	-	75%	-
EXTENSION OF INFLAMMATORY INFILTRATE				
(1) absence of inflammation	-	50%	-	50%
(2) partial extension of connective tissue	-	50%	-	50%
(3) entire extension of connective tissue, without reaching bone tissue	100%	-	25%	-
(4) entire extension of connective tissue and bone tissue	-	-	75%	-
EXTERNAL RADICULAR RESORPTION (CEMENTUM AND DENTIN)				
(1) absence	-	-	-	-
(2) only inactive reabsorption areas	-	50%	-	50%
(3) minor active reabsorption areas	75%	50%	25%	50%
(4) several active reabsorption areas	25%	-	75%	-
ALVEOLAR BONE RESORPTION				
(1) within normality patterns	-	-	-	-
(2) small amount of resorption bone areas	-	100%	-	100%
(3) moderate amount of resorption bone areas	75%	-	25%	-
(4) large amount of resorption bone areas	25%	-	75%	-
CELLULAR PATTERN AND CONNECTIVE TISSUE STRUCTURE				
(1) moderate number of fibroblasts and large amount of collagen fibres (dense connective tissue)	-	25%	-	-
(2) moderate amount of both fibroblasts and collagen fibres	-	75%	-	100%
(3) small amount of both fibroblasts and collagen fibres	100%	-	100%	-
(4) severe tissue disorganisation with necrosis areas	-	-	-	-
PATTERN OF STRUCTURATION OF THE BONE ALVEOLAR				
(1) bone trabeculae with regular contours coated with active osteoblasts, including areas of new bone formation	-	-	-	-
(2) bone trabeculae with irregular contours coated with active osteoblasts and osteoclasts	-	100%	-	100%
(3) bone trabeculae with irregular contours coated with active osteoclasts	100%	-	100%	-
(4) partial tissue breakdown with areas of bone necrosis	-	-	-	-

**Table 2:** The parameters, scores, and distribution of specimens based on the histopathological analysis of the intestines of the experimental groups.

HISTOPATHOLOGICAL ANALYSES				
PARAMETERS AND SCORES	PERCENTAGE OF SPECIMENS			
	EXPERIMENTAL GROUPS			
	LIP	LIP + RPI	LIP + DIC	LIP + DIC + RPI
CELLULARITY PATTERN OF THE INTESTINAL MUCOSAL EPITHELIUM				
(1) full integrity of epithelial tissue	100%	100%	-	-
(2) punctual foci of discontinuity of the epithelial tissue (commitment <10% of the circumference of the intestinal mucosa)	-	-	75%	100%
(3) punctual foci of discontinuity of the epithelial tissue (commitment >10% and <20% of the circumference of the intestinal mucosa)	-	-	25%	-
(4) large areas of discontinuity of the epithelial tissue (commitment >20% of the circumference of the intestinal mucosa)	-	-	-	-
INFLAMMATORY INFILTRATE PRESENT IN THE LAMINA PROPRIA				
(1) presence of rare inflammatory cells, compatible with the absence of inflammation	100%	100%	-	-
(2) presence of a small number of inflammatory cells (up to 1/3 of the cells are inflammatory)	-	-	25%	100%
(3) presence of a moderate number of inflammatory cells (1/3 to 2/3 of the cells are inflammatory	-	-	75%	-
(4) presence of a large number of inflammatory cells (more than 2/3 of the cells are inflammatory)	-	-	-	-
EXTENSION OF THE INFLAMMATORY PROCESS IN THE INTESTINAL WALL				
(1) absence of inflammation	100%	100%	-	-
(2) reaching exclusively the intestinal mucosa	-	-	100%	100%
(3) reaching the mucosa and submucosa of the intestine	-	-	-	-
(4) reaching the mucosa, submucosa, muscularis, and serosa of the intestine	-	-	-	-

## Data Availability

The data presented in this study are available upon request from the corresponding author.
